# Acute and chronic hypopituitarism following traumatic brain injury: a systematic review and meta-analysis

**DOI:** 10.1007/s10143-024-03088-3

**Published:** 2024-11-11

**Authors:** Ghaith S. Aljboor, Aoun Tulemat, Ali Ridha Al-Saedi, Mugurel Petrinel Radoi, Corneliu Toader, Toma Marius Papacocea

**Affiliations:** 1 Department of Neurosurgery. 020021, University of Medicine and Pharmacy “Carol Davila”, Bucharest, Romania; 2 Neurosurgical Department, . Pantelimon Emergency Hospital, Bucharest, Romania; 3 Department of Neurosurgery, National Institute of Neurology and Neurovascular Diseases, 020021 Bucharest, Romania

**Keywords:** Traumatic brain injury, Pituitary axis dysfunction, Prevalence, Meta-analysis, Temporal patterns, Endocrine abnormalities

## Abstract

Traumatic brain injury (TBI) is associated with various endocrine abnormalities, including pituitary axis dysfunction. Understanding the prevalence and temporal patterns of these dysfunctions is crucial for effective clinical management. This study aimed to systematically review the literature and conduct a meta-analysis to determine the prevalence of pituitary axis dysfunction following TBI, assess temporal patterns across different post-injury durations, and identify potential contributing factors. A comprehensive search was conducted across multiple electronic databases between 1st of January 2000 until 31st March 2024. Studies reporting the prevalence of pituitary axis dysfunction post-TBI were included. Pooled estimates with 95% confidence intervals (CIs) were calculated using random-effects models in the R statistical software. Subgroup analyses were performed based on duration post-TBI (< 3 months, 3–6 months, 6–12 months, > 12 months) to explore temporal variations. Heterogeneity was assessed using the I^2 statistic. A total of 52 studies were included in the meta-analysis, encompassing 7367 participants. The pooled estimate for the prevalence of any pituitary axis dysfunction post-TBI was 33% (95% CI [28%; 37%]). Subgroup analysis by duration revealed varying prevalence rates: < 3 months (40%, 95% CI [27%; 53%]), 3–6 months (31%, 95% CI [15%; 47%]), 6–12 months (26%, 95% CI [19%; 33%]), and > 12 months (32%, 95% CI [26%; 38%]). Prevalence of multiple axes affection was 7% (95% CI [6%; 9%]), with varying rates across durations. Specific axes affection varied: Growth Hormone (GH) deficiency was 18% (95% CI [14%; 21%]), adrenocorticotropic hormone (ACTH) deficiency was 10% (95% CI [8%; 13%]), pituitary–gonadal axis hormones deficiency was 16% (95% CI [12%; 19%]), and thyroid-stimulating hormone (TSH) deficiency was 6% (95% CI [5%; 7%]). This meta-analysis highlights a significant prevalence of pituitary axis dysfunction following TBI, with temporal variations observed across different post-injury durations. The findings underscore the importance of tailored clinical management strategies based on the duration and type of dysfunction. Further research addressing potential contributing factors is warranted to enhance understanding and management of these conditions.

## Background

Traumatic brain injury (TBI) is a significant public health concern globally, contributing to substantial morbidity and mortality rates across all age groups [1, 2]. TBI encompasses a spectrum of injuries resulting from external mechanical forces to the head, leading to transient or permanent neurological dysfunction. Common causes of TBI include motor vehicle accidents, falls, assaults, and sports-related injuries [3–5].

One of the lesser-known but clinically significant consequences of TBI is its potential to disrupt the functioning of the pituitary gland, leading to a spectrum of endocrine abnormalities collectively termed hypopituitarism [6–8]. Hypopituitarism following TBI can manifest as deficiencies in the adenohypophysis hormones such as: growth hormone (GH), adrenocorticotropic hormone (ACTH), thyroid-stimulating hormone (TSH), and pituitary–gonadal axis hormones. Furthermore, it can also manifest as deficiencies in the neurohypophysis hormones such as: antidiuretic hormone (ADH) [8, 9].

The pathophysiology of pituitary dysfunction post-TBI is multifactorial and not entirely understood. Direct trauma to the pituitary gland, disruption of the hypothalamic-pituitary axis, ischemic injury, and neuroinflammatory responses are among the proposed mechanisms contributing to post-TBI hypopituitarism [10]. The extent and severity of pituitary dysfunction may vary depending on factors such as the nature of the injury (e.g., focal vs. diffuse), TBI severity (mild, moderate, severe), time elapsed since the injury, and individual patient characteristics [11].

Understanding the prevalence and patterns of pituitary axis dysfunction following TBI is essential for several reasons. Firstly, unrecognized and untreated hypopituitarism can lead to a range of adverse health outcomes, including metabolic derangements, impaired quality of life, cognitive deficits, and increased mortality rates [12]. Secondly, early detection and management of hormone deficiencies can mitigate long-term complications and improve patient outcomes. However, diagnosing post-TBI hypopituitarism presents challenges due to its nonspecific symptoms, overlapping with those of TBI sequelae and other comorbidities [9, 13].

Previous epidemiological studies investigating the prevalence of hypopituitarism following TBI have reported varying prevalence rates, ranging from single-digit percentages to more than 50%, this variation in prevalence might be related to varying TBI severity, which is established by Glasgow Coma Score (GCS) and paraclinical findings, and depending on the study population, methodology, and diagnostic criteria employed [6, 14]. However, a comprehensive synthesis and analysis of existing literature are necessary to provide a more accurate estimate of the prevalence of pituitary axis dysfunction post-TBI, identify potential risk factors associated with its development, and guide clinical management strategies.

## Study aim and objectives

The aim of this study is to conduct a systematic review and meta-analysis to determine the prevalence of pituitary axis dysfunction following traumatic brain injury (TBI) and to explore temporal trends in prevalence rates over different time intervals. By the following:

1. Assess the prevalence of pituitary axis dysfunction after traumatic brain injury (TBI).
2. Examine different types of hormone deficiencies related to the pituitary gland post-injury.
3. Identify temporal trends in the prevalence of pituitary dysfunction over various time frames, from less than 3 months to over 12 months after the injury.

## Methodology

### Study design

This meta-analysis follows a systematic and comprehensive approach to synthesize available evidence on the prevalence of hypopituitarism following traumatic brain injury (TBI). The study design adhered to the Preferred Reporting Items for Systematic Reviews and Meta-Analyses (PRISMA) guidelines [15].

### Study duration

The meta-analysis includes studies published between 1st of January 2000 until the search date of 31st of March 2024. No restrictions were placed on the publication year to ensure the inclusion of relevant studies spanning a wide timeframe.

### Search strategy

A comprehensive literature search was conducted across multiple electronic databases, including PubMed, Web of Science, Scopus, Medline, the Cochrane Library, and Google Scholar. The search strategy utilized a combination of terms: *traumatic brain injury, pituitary axis dysfunction, hypopituitarism, endocrine abnormalities, prevalence, and epidemiology*. Extracted articles from google scholar were vetted by our authors through screening the title and abstract without selecting articles reporting exclusively endocrine findings. Boolean operators (AND, OR) were used to refine the search and ensure comprehensive coverage of relevant literature. The search was limited to studies published in English between 1st of January 2000 until the search date of 31st of March 2024.

### Study selection

**Fig. 1:**
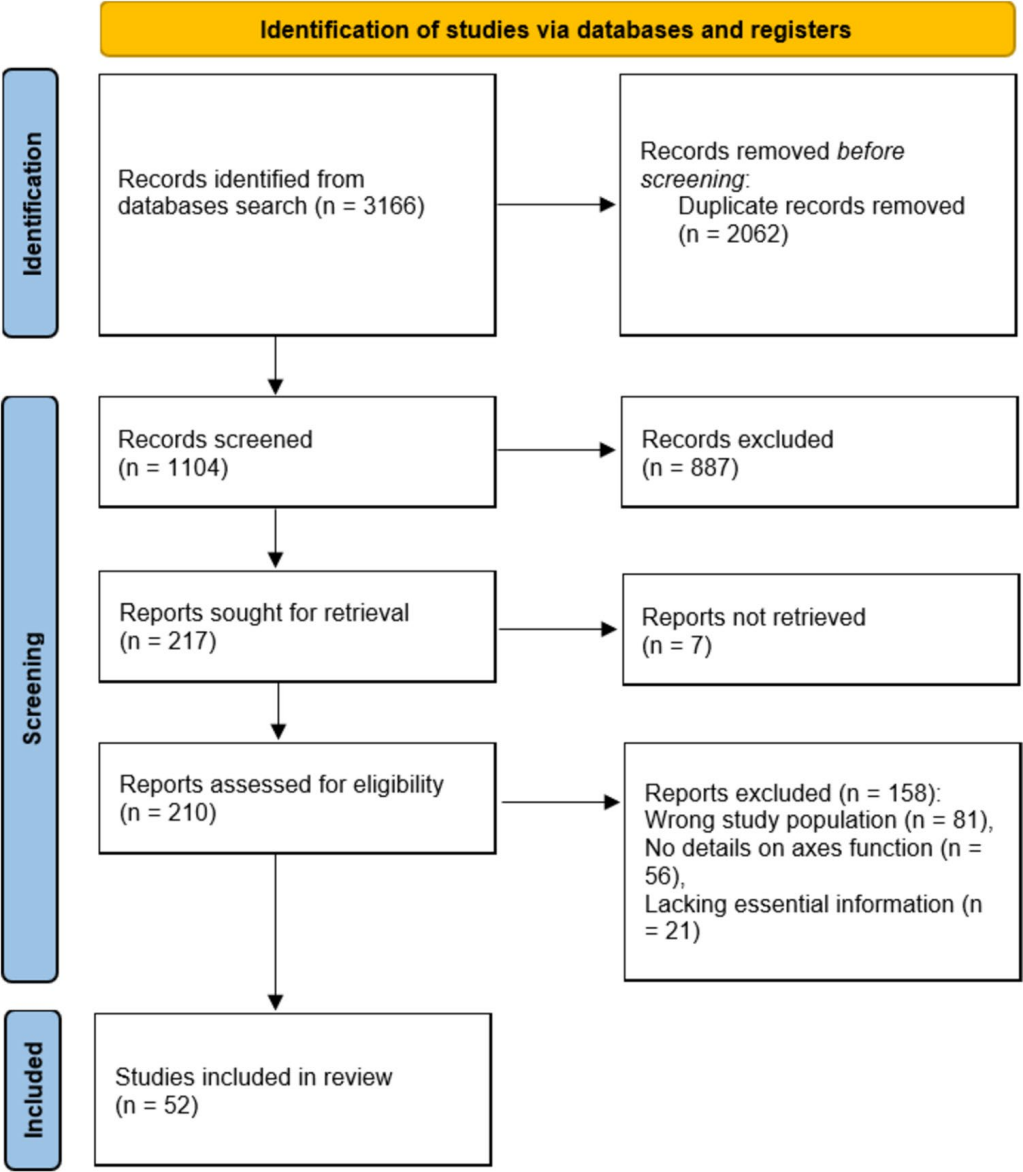
PRISMA flow diagram for summary of search and screening processes

Studies were initially screened based on titles and abstracts to identify potentially relevant articles by three authors (GA, AT, ARA) independently. Reviewers avoided bias by disregarding authors’ name and affiliated institutions. Subsequently, full-text articles were retrieved for detailed evaluation against the inclusion criteria. The outcome was then gathered into Microsoft Excel and the Preferred Reporting Items for Systemic Reviews and Meta-Analysis (PRISMA) statement was performed in its three stages (Fig. 1). Consensus was the method to resolve disagreement.

### Inclusion criteria

Studies were included if they met the following criteria:

1. Observational studies, cohort studies, case–control studies, and cross-sectional studies.
2. Published in English.
3. Pituitary dysfunction post traumatic brain injury diagnosed by blood tests using cutoff values based current guidelines.
4. Reported prevalence rates or provided data to calculate prevalence.
5. Used clear diagnostic criteria for pituitary dysfunction.
6. Included adult human subjects.

### Exclusion criteria

Studies were excluded if they:

1. Did not report primary data on pituitary dysfunction prevalence regardless of the study design.
2. Secondary sources (i.e. not reporting original data)
3. Were duplicates or redundant publications.
4. Had sample sizes of fewer than 10 participants to ensure statistical reliability.
5. Studies with a score bellow 7 on Newcastle–Ottawa Scale (NOS)

### Data extraction and synthesis

Data extraction was performed independently by two reviewers using a standardised data extraction form. Extracted data included study characteristics (e.g., author names, publication year, study design, country), participant demographics (e.g., sample size, age, gender distribution), TBI severity, prevalence rates of any pituitary axis dysfunction and specific axis dysfunctions (e.g., growth hormone deficiency, adrenocorticotropic hormone deficiency), and temporal data (e.g., duration post-TBI).

### Quality assessment

The methodological quality of included studies was assessed using the Newcastle–Ottawa Scale (NOS) for observational studies. The NOS evaluates studies based on three domains: selection of study groups, comparability of groups, and ascertainment of outcomes. Studies were evaluated by three investigators independently on a scale of 0 to 9, with higher scores indicating higher methodological quality [16]. Studies with a score between 7 and 9 were included.

### Software and statistical analysis

The R meta package was utilised for statistical analyses [17]. Pooled estimates of prevalence rates were calculated using random-effects models, which account for both within-study and between-study variability. The most used method with random-effects model is the DerSimonian-Laird (D-L) method which was employed to estimate the between-study variance (tau-squared). D-L method was applied due its simplicity and computational efficiency in which makes it reliable for diverse levels of heterogeneity as seen in our data. The publication bias was assessed using funnel plots. Forest plots were generated to visually represent the pooled estimates along with their 95% confidence intervals (CIs).

### Assessment of heterogeneity

Heterogeneity among studies was assessed using the I^2 statistic, with values of 25%, 50%, and 75% indicating low, moderate, and high heterogeneity, respectively. Subgroup analyses were conducted based on the duration post-TBI (< 3 months, 3–6 months, 6–12 months, > 12 months) to explore temporal variations in prevalence rates.

## Results

### Search results

Our comprehensive search across multiple databases including PubMed, Web of Science, Scopus, Medine, the Cochrane Library, and Google Scholar yielded a total of 3166 records. After removing duplicates (2062 records), 1104 unique records were screened based on their titles and abstracts. Of these, 887 records were excluded, leading to 217 studies being sought for full-text retrieval. However, 7 studies were not retrieved, resulting in 210 studies that were assessed for eligibility. Through a meticulous eligibility assessment, 158 studies were further excluded, leaving us with a final inclusion of 52 studies for our meta-analysis (Fig. 1). The characters of the included studies are presented in Table 1.

**Table 1:** Characters of the included studies and populations of TBI patients (*n* = 52)

Study	Country	Study design	Sample size	M%	Age	Time after event, months	BMI	Mild TBI, n	Moderate TBI, n	Severe TBI, n	NOS Score
Agha et al., 2005 [18]	Ireland	Prospective	50	76.0%	37 ± 14	7d, 6 m, 12 m	NR	NR	NR	NR	8
Agha et al., 2004 [19]	Ireland	Cross-sectional	102	83.3%	28 (15–65)	6-36 m	NR	NR	NR	NR	8
Aimaretti et al., 2004 [20]	Italy	Prospective	100	69.0%	37.1 ± 1.8	3 m	23.7 ± 0.4	55	24	21	9
Aimaretti et al., 2005 [21]	Italy	Prospective	70	71.4%	39.3 ± 2.4	12 m	23.8 ± 0.4	33	22	15	8
Alavi et al., 2015 [22]	UK	Prospective	105	74.3%	NR	6-12 m, > 12 m	NR	NR	NR	NR	9
Bavisetty et al., 2008 [23]	USA	Prospective	70	81.4%	32	6-9 m	NR	NR	NR	NR	8
Bensalah et al., 2020 [24]	Algeria	Prospective	133	96.2%	32.2 ± 10	3 m, 12 m	NR	0	100	33	8
Berg et al., 2010 [25]	Germany	Cross-sectional	246	54.1%	39 ± 14	4-47 m	25.8 ± 4.2	NR	NR	NR	8
Bondanelli et al., 2004 [26]	Italy	Cross-sectional	50	80.0%	37.6 ± 2.4	12-64 m	24.6 ± 0.4	16	7	27	8
Bushnik et al., 2007 [27]	USA	Cross-sectional	64	67.2%	42 ± 12	1.2-31y	NR	NR	NR	NR	8
Choudhary et al., 2023 [28]	India	Prospective	100	NR	NR	2d, 14d, 1 m, 3 m, 6 m	NR	0	77	23	9
Ciarlone et al., 2020 [29]	USA	Retrospective	59	94.9%	NR	1y, 2y, 3y	24.5 ± 3.6	59	0	0	7
Claessen et al., 2024 [30]	Iceland	Retrospective	131	0.0%	29.3 ± 7.6	4.3y	26.3 ± 4.7	131	0	0	7
Cuesta et al., 2016 [31]	Ireland	Prospective	112	68.8%	31 ± 11	19 m	NR	0	112		8
Daloglu et al., 2024 [32]	Turkey	Retrospective	30	86.7%	38.1 ± 14.2	> 12 m	26.4 (23.0–27.9)	NR	NR	NR	7
Dimopoulou et al., 2004 [33]	Greece	Prospective	34	79.4%	37 ± 16	9-60d	NR	0	9	25	8
Frendl et al., 2017 [34]	Hungary	Prospective	61	75.4%	44 ± 19	6-12 m	NR	61	0	0	8
Gupta et al., 2021 [35]	India	Prospective	84	66.7%	40.2 (18–78)	1d, 7d	NR	36	34	14	8
Herrmann et al., 2006 [36]	Germany	Cross-sectional	76	69.7%	39 ± 14	5-47 m	25.8 ± 4.2	NR	NR	NR	8
High et al., 2010 [37]	USA	Cross-sectional	83	NR	NR	> 1y	NR	NR	NR	NR	8
Jeong et al., 2010 [38]	Korea	Prospective	65	70.8%	30.1	6 m	NR	NR	NR	NR	8
Kleindienst et al., 2009 [39]	Germany	Prospective	71	80.3%	53 ± 20	7d, 2y	25.6 ± 3.9	24	32	15	7
Klose et al., 2007 [40]	Denmark	Cross-sectional	104	75.0%	37 (21–64)	10-27 m	24.1 (19.2–30.0)	44	20	40	9
Kokshoorn et al., 2011 [41]	Netherlands	Cross-sectional	112	67.0%	48 (19–69)	Mean 4.2y	26.7 ± 4.8	64	48		9
Kopczak et al., 2014 [42]	Germany	Cross-sectional	340	75.6%	40 ± 15	Median 5-12w	23.1 ± 3.9	NR	NR	NR	7
Krahulik et al., 2010 [43]	Czech Republic	Prospective	89	74.2%	36 (18–65)	6 m, 1y	NR	NR	NR	NR	8
Krewer et al., 2016 [44]	Germany	Prospective	245	67.3%	50	> 1 year (1–55 years)	26.4 ± 5.4	51	6	37	8
Kumar et al., 2016 [45]	India	Prospective	56	78.6%	31.7 ± 7.8	0-10d, 6 m, 12 m	NR	6	19	31	8
Leal-Cerro et al., 2005 [46]	Spain	Cross-sectional	170	58.2%	29.2 ± 1.1	> 12 m	24.7 ± 0.2	NR	NR	NR	7
Lee et al., 2021 [47]	USA	Retrospective	58	86.2%	41 (34–45)	96 m	29.9 (26.8–35.0)	42	10	6	8
Monreau et al., 2012 [48]	France	Prospective	55	83.6%	36.1 ± 11.3	79.2 m	25.2 (18.0–35.5)	NR	NR	NR	8
Nemes et al., 2016 [49]	Hungary	Prospective	63	82.5%	37.5 ± 17	1.1y	NR	0	0	63	7
Nourollahi et al., 2014 [50]	Germany	Retrospective	97	70.1%	33.5 ± 14.4	12-64 m	NR	34	8	45	9
Park et al., 2010 [51]	Korea	Prospective	45	71.1%	32.6 ± 17.6	16.4 m	NR	0	19	26	9
Popovic et al., 2004 [52]	Serbia	Cross-sectional	67	58.2%	37.5 ± 1.8	1-22y	24.8 ± 0.5	NR	NR	NR	7
Prodam et al., 2013 [53]	Italy	Retrospective	54	70.4%	37.1 ± 2.5	7.4y	24.1 ± 1.1	0	9	45	9
Salleh et al., 2023 [54]	Malaysia	Prospective	105	79.0%	36.9 ± 12.4	9.8 m	NR	36	21	48	9
Schneider et al., 2011 [55]	Germany	Cross-sectional	825	73.5%	45.1 ± 29.1	3 m, 5 m	27.2 ± 9.8	NR	NR	NR	8
Schneider et al., 2006 [56]	Germany	Prospective	78	66.7%	36 ± 15	3 m, 12 m	22.0 ± 3.1	NR	NR	NR	8
Sigurjónsson et al., 2022 [57]	Iceland	Prospective	21	81.0%	34	0-6d	NR	0	6	15	9
Silva et al., 2015 [58]	USA	Retrospective	165	70.9%	41.6 (18,076)	40.4 m	NR	114	51		8
Srinivasan et al., 2009 [59]	USA	Cross-sectional	18	66.7%	31.9 (20–59)	Mean 8 m	26.7 (22.3–34.8)	NR	NR	NR	8
Tanriverdi et al., 2013 [60]	Turkey	Prospective	25	80.0%	36.8 ± 2.1	5y	NR	16	5	4	8
Tanriverdi et al., 2006 [61]	Turkey	Prospective	52	82.7%	35.9 ± 13.8	1d, 12 m	NR	33	8	13	9
Tanriverdi et al., 2008 [62]	Turkey	Prospective	30	83.3%	37.2 ± 2.4	3y	NR	19	6	5	8
Tölli et al., 2015 [63]	Sweden	Prospective	84	77.4%	48.3 ± 16.9	10 days	25.5 ± 4.9	0	21	63	8
Tölli et al., 2017 [64]	Sweden	Prospective	56	73.2%	47.1 ± 16.6	10d, 3 m, 6 m, 12 m	25.6 ± 4.8	0	12	44	9
Ulfarsson et al., 2010 [65]	Sweden	Retrospective	51	74.5%	37.9 (16–64)	68 m	24.0 (18–29)	NR	NR	NR	8
van der Eerden et al., 2010 [66]	Netherlands	Cross-sectional	107	65.4%	45 (22–63)	3-30 m	NR	77	30		8
Verma et al., 2021 [67]	India	Cross-sectional	200	87.5%	NR	3 m- > 5y	NR	0	0	200	8
Zacharia et al., 2022 [68]	India	Prospective	66	100.0%	32.9 ± 5.8	6-24y	24.7 ± 4.2	0	30	36	9
Zgarljardic et al., 2011 [69]	USA	Retrospective	138	71.0%	35.8 ± 10.7	63.4 m	NR	NR	NR	NR	8

## Characteristics of the included studies

### Study design

The included studies varied in their design, encompassing prospective 28 (53.8%), retrospective 9 (17.4%), and cross-sectional 15 (28.8%) approaches. Prospective studies such as Agha et al. (2005), Aimaretti et al. (2004, 2005), and others provided longitudinal insights into acute and chronic hypopituitarism following traumatic brain injury (TBI) [18, 20, 21]. Retrospective studies like Srinivasan et al. (2009) and Nourollahi et al. (2014) contributed retrospective data analysis, while cross-sectional studies such as Berg et al. (2010) offered a snapshot of the condition across different time points [25, 50, 59].

### Country and population

The geographical distribution of the studies spanned various countries including Ireland, Italy, the UK, the USA, Germany, India, and others. Studies from the mentioned regions reported varying prevalence rates of hypopituitarism post-TBI. Studies like Klose et al. (2007) in Denmark and Tanriverdi et al. (2013, 2006, 2008) in Turkey highlighted the global impact of this condition [40, 60–62].

### Patient demographics

The sample sizes across studies ranged widely, from smaller cohorts like Daloglu et al. (2024) with 30 participants to larger studies like Schneider et al. (2011) with 825 participants [32, 55]. The mean age of participants varied from 29.2 years (Leal-Cerro et al., 2005) to 48.3 years (Tölli et al., 2015) [46, 63]. Male proportions in the studies ranged from 0% (Claessen et al., 2024) to 100% (Zacharia et al., 2022), reflecting the gender distribution within each cohort [30, 68].

### Quality assessment (NOS)

Quality assessment using the Newcastle–Ottawa Scale (NOS) revealed scores ranging from 7 to 9 with an average of 8.09 across the included studies. The three domains, selection of study groups, comparability of groups, and ascertainment of outcomes, averaged 3.9, 1.39, and 2.8, respectively. Studies like Choudhary et al. (2023) and Tanriverdi et al. (2006, 2008) obtained NOS scores of 9, indicating a high level of methodological quality, while others obtained scores of 7 or 8, reflecting robustness in study design and execution [28, 61, 62].

### Quantitative data synthesis

#### Prevalence of any and multiple axes affection

**Fig. 2:**
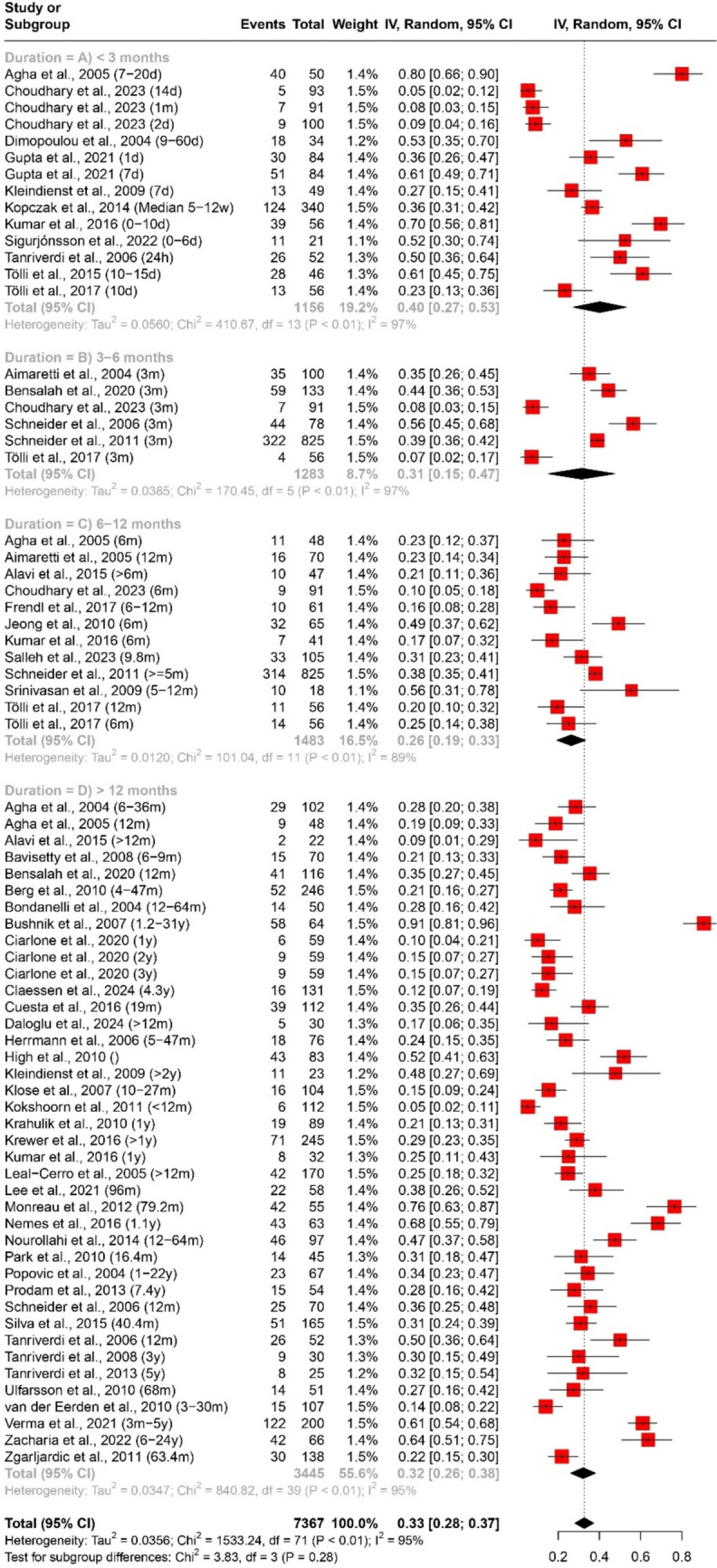
Forest plot of the pooled prevalence of any axis affection (*n* = 52)

**Fig. 3:**
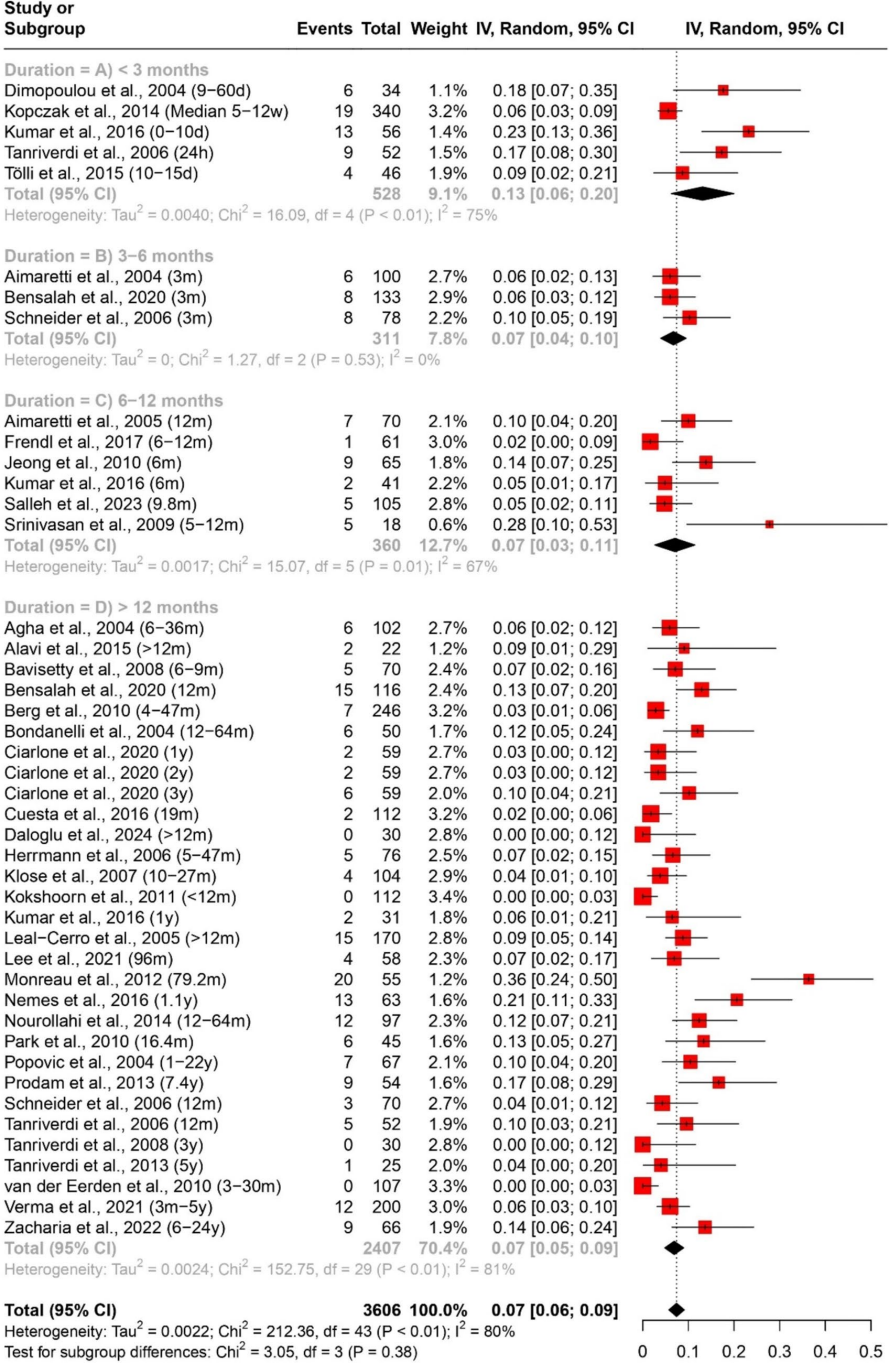
Forest plot of the pooled prevalence of multiple axes affection (*n* = 36)

The prevalence of any and multiple axes affection following traumatic brain injury (TBI) was assessed through a quantitative data synthesis. A random-effects model, along with subgroup analysis and a test for subgroup differences, was used to address heterogeneity. The results are presented in Table 2 and Figs. 2 and 3.

**Table 2:** Pooled effect sizes and heterogeneity assessment for the prevalence of any and multiple axes affection outcomes

Measurement	Duration	Number of datasets	Number of studies	Participants (Total)	Model	Pooled Estimate [95% CI]	Heterogeneity (
Prevalence of any axis affection (Fig. 2)	< 3 months (Fig. 2A)	14	11	1156	Random Effects	40% [27%; 53%]	Tau^2 = 0.0560; Chi^2 = 410.67, df = 13 (*P* < 0.01); I^2 = 97%
	3–6 months (Fig. 2B)	6	6	1283	Random Effects	31% [15%; 47%]	Tau^2 = 0.0385; Chi^2 = 170.45, df = 5 (*P* < 0.01); I^2 = 97%
	6–12 months (Fig. 2C)	12	11	1483	Random Effects	26% [19%; 33%]	Tau^2 = 0.0120; Chi^2 = 101.04, df = 11 (*P* < 0.01); I^2 = 89%
	> 12 months (Fig. 2D)	40	38	4120	Random Effects	32% [26%; 38%]	Tau^2 = 0.0347; Chi^2 = 840.82, df = 39 (*P* < 0.01); I^2 = 95%
	Overall	72	52	7367	Random Effects	33% [28%; 37%]	Tau^2 = 0.0356; Chi^2 = 1533.24, df = 71 (*P* < 0.01); I^2 = 95%
Prevalence of multiple axes affection (Fig. 3)	< 3 months (Fig. 3A)	5	5	528	Random Effects	13% [6%; 20%]	Tau^2 = 0.0040; Chi^2 = 16.09, df = 4 (*P*< 0.01); I^2 = 75%
	3–6 months (Fig. 3B)	3	3	311	Random Effects	7% [4%; 10%]	Tau^2 = 0; Chi^2 = 1.27, df = 2 (*P* = 0.53); I^2 = 0%
	6–12 months (Fig. 3C)	6	6	360	Random Effects	7% [3%; 11%]	Tau^2 = 0.0017; Chi^2 = 15.07, df = 5 (*P* = 0.01); I^2 = 67%
	> 12 months (Fig. 3D)	30	28	2407	Random Effects	7% [5%; 9%]	Tau^2 = 0.0024; Chi^2 = 152.75, df = 29 (*P* < 0.01); I^2 = 81%
	Overall	44	36	3606	Random Effects	7% [6%; 9%]	Tau^2 = 0.0022; Chi^2 = 212.36, df = 43 (*P* < 0.01); I^2 = 80%

#### Prevalence of any axis affection

The analysis included data from 72 datasets and 52 studies, encompassing a total of 7367 participants. The pooled estimate for the prevalence of any axis affection was 33% (95% CI [28%; 37%]), indicating a substantial occurrence of pituitary axis dysfunction post-TBI.

Subgroup analysis based on duration post-TBI revealed varying prevalence rates. Within the first 3 months (< 3 months), the prevalence was highest at 40% (95% CI [27%; 53%]), as illustrated in Fig. 2A. This prevalence gradually decreased over time: 31% (95% CI [15%; 47%]) at 3–6 months (Fig. 2B), 26% (95% CI [19%; 33%]) at 6–12 months (Fig. 2C), and 32% (95% CI [26%; 38%]) beyond 12 months (Fig. 2D). Overall, substantial heterogeneity was observed across all durations, with I^2 values ranging from 89 to 97%.

#### Prevalence of multiple axes affection

The analysis also investigated the prevalence of multiple axes affection, indicating dysfunction across multiple pituitary axes simultaneously. Across different durations, the overall prevalence of multiple axes affection was 7% (95% CI [6%; 9%]), as depicted in Fig. 3.

Subgroup analysis by duration revealed varying prevalence rates: 13% (95% CI [6%; 20%]) for < 3 months (Fig. 3A), 7% (95% CI [4%; 10%]) for 3–6 months (Fig. 3B), 7% (95% CI [3%; 11%]) for 6–12 months (Fig. 3C), and 7% (95% CI [5%; 9%]) for > 12 months (Fig. 3D). Notably, heterogeneity levels varied across these subgroups, ranging from 0 to 81%.

The observed heterogeneity suggests that factors beyond duration, such as TBI severity, patient demographics, and study methodologies, may contribute to the variability in prevalence rates across different axes affection and timeframes.

#### Prevalence of specific axes affection

**Fig. 4:**
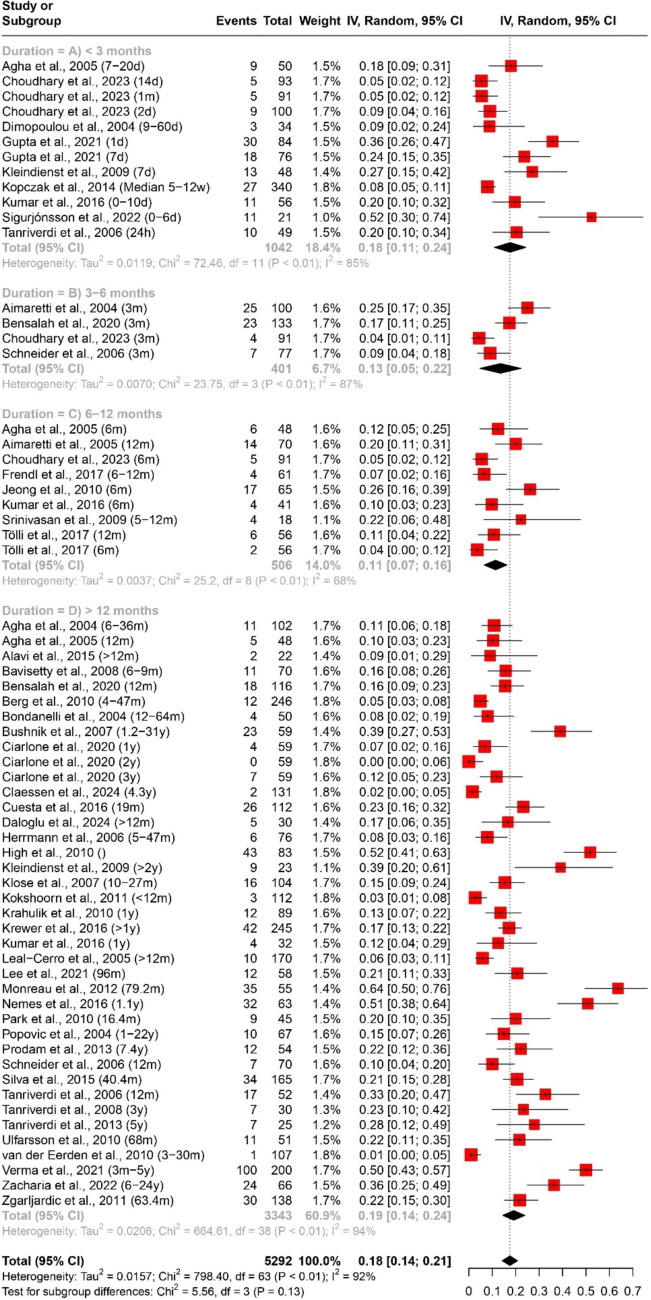
Forest plot of the pooled prevalence of growth hormone deficiency (*n* = 46)

**Fig. 5:**
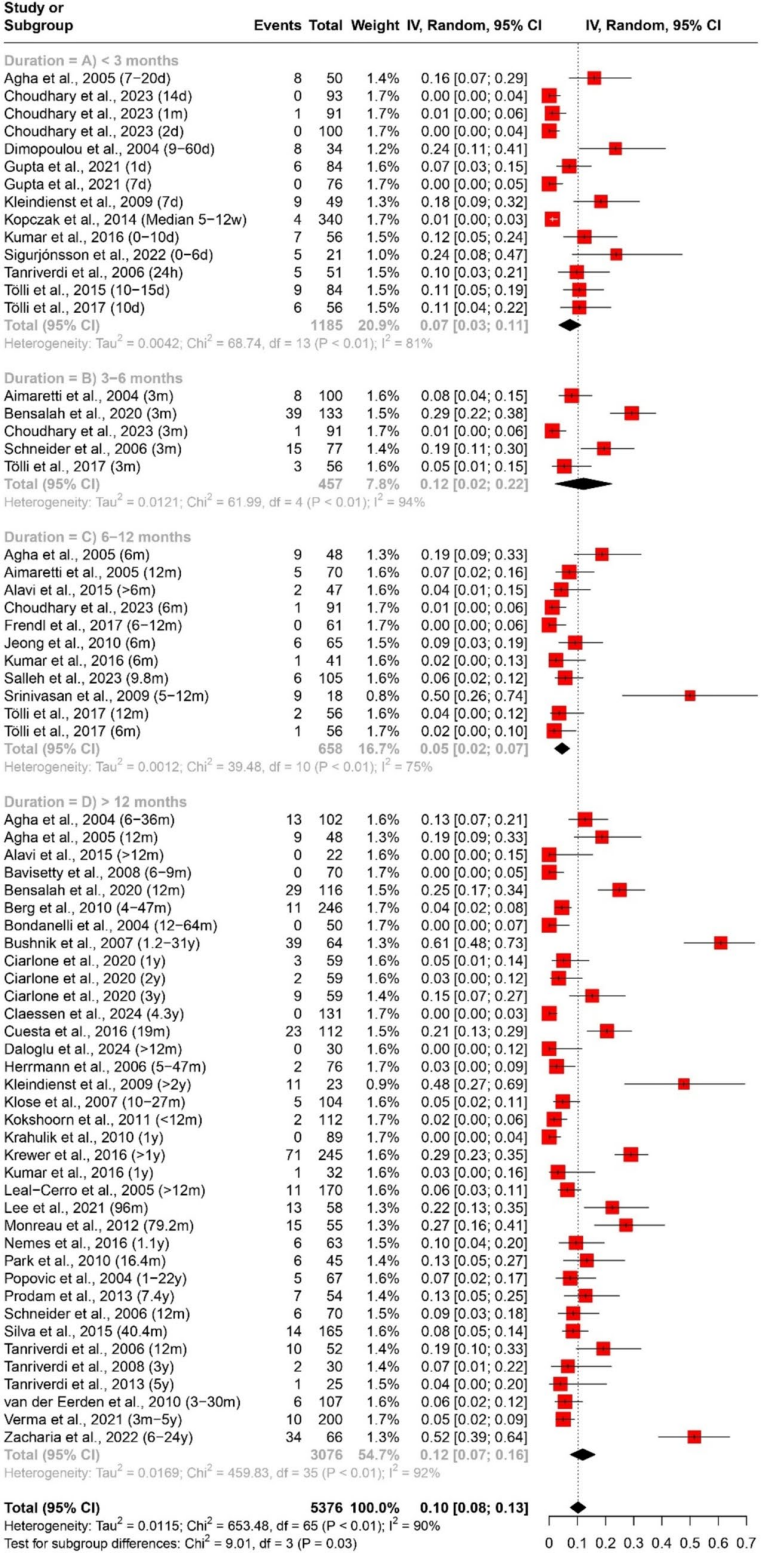
Forest plot of the pooled prevalence of adrenocorticotrophic hormone deficiency (*n* = 47)

**Fig. 6:**
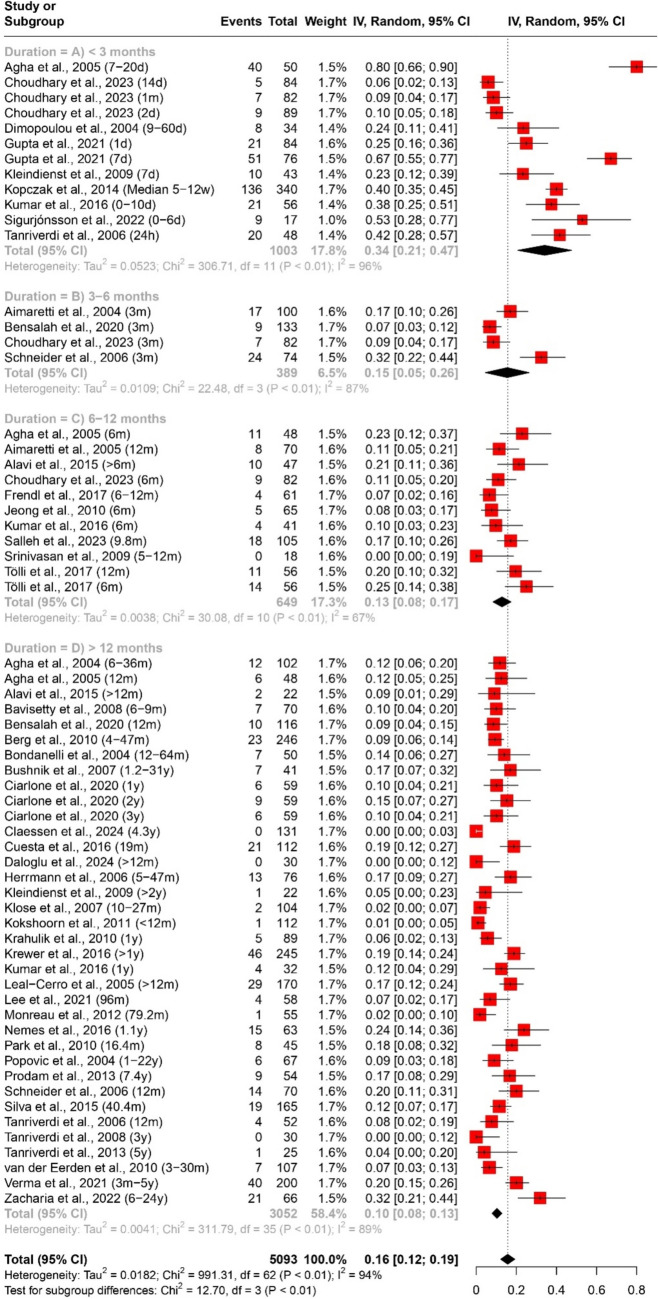
Forest plot of the pooled prevalence of pituitary–gonadal axis hormones deficiency (*n* = 45)

**Fig. 7:**
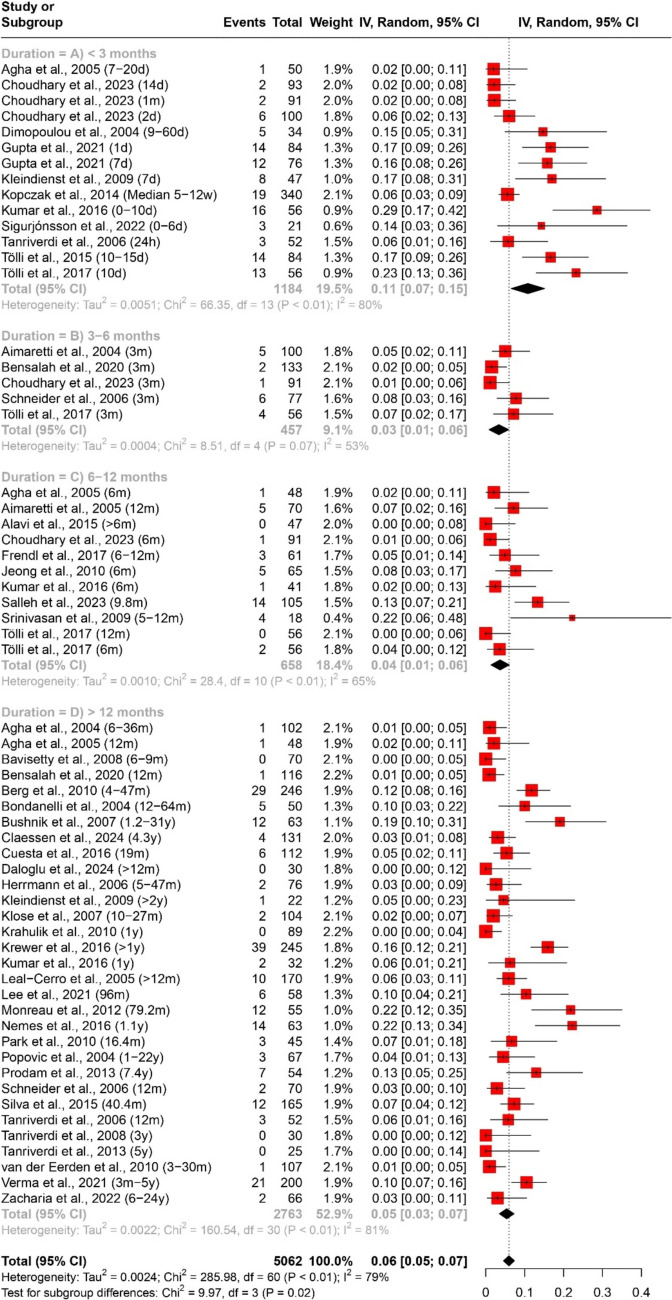
Forest plot of the pooled prevalence of TSH deficiency (*n* = 45)

In evaluating the prevalence of specific axes affection following traumatic brain injury (TBI), a quantitative data synthesis was conducted. We used a random-effects model, accompanied by subgroup analysis and a test for subgroup differences to address heterogeneity, as outlined in Table 3 and Figs. 4, 5, 6, 7.

**Table 3:** Pooled effect sizes and heterogeneity assessment for the prevalence of specific axes affection outcomes

Measurement	Duration	Number of datasets	Number of studies	Participants (Total)	Model	Pooled Estimate [95% CI]	Heterogeneity
Prevalence of GH deficiency (Fig. 4)	< 3 months (Fig. 4A)	12	9	1042	Random Effects	18% [11%; 24%]	Tau^2 = 0.0119; Chi^2 = 72.46, df = 11 (*P* < 0.01); I^2 = 85%
	3–6 months (Fig. 4B)	4	4	401	Random Effects	13% [5%; 22%]	Tau^2 = 0.0070; Chi^2 = 23.75, df = 3 (*P* < 0.01); I^2 = 87%
	6–12 months (Fig. 4C)	9	8	506	Random Effects	11% [7%; 16%]	Tau^2 = 0.0037; Chi^2 = 25.2, df = 8 (*P* < 0.01); I^2 = 68%
	> 12 months (Fig. 4D)	39	37	3343	Random Effects	19% [14%; 24%]	Tau^2 = 0.0206; Chi^2 = 664.61, df = 38 (*P* < 0.01); I^2 = 94%
	Overall	64	46	5292	Random Effects	18% [14%; 21%]	Tau^2 = 0.0157; Chi^2 = 798.40, df = 63 (*P* < 0.01); I^2 = 92%
Prevalence of ACTH deficiency (Fig. 5)	< 3 months (Fig. 5A)	14	11	1185	Random Effects	7% [3%; 11%]	Tau^2 = 0.0042; Chi^2 = 68.74, df = 13 (*P* < 0.01); I^2 = 81%
	3–6 months (Fig. 5B)	5	5	457	Random Effects	12% [2%; 22%]	Tau^2 = 0.0121; Chi^2 = 61.99, df = 4 (*P* < 0.01); I^2 = 94%
	6–12 months (Fig. 5C)	11	10	658	Random Effects	5% [2%; 7%]	Tau^2 = 0.0012; Chi^2 = 39.48, df = 10 (*P* < 0.01); I^2 = 75%
	> 12 months (Fig. 5D)	36	34	3076	Random Effects	12% [8%; 16%]	Tau^2 = 0.0169; Chi^2 = 459.83, df = 35 (*P* < 0.01); I^2 = 92%
	Overall	66	47	5376	Random Effects	10% [8%; 13%]	Tau^2 = 0.0115; Chi^2 = 653.48, df = 65 (*P*< 0.01); I^2 = 90%
Prevalence of pituitary–gonadal axis hormones deficiency (Fig. 6)	< 3 months (Fig. 6A)	12	9	1003	Random Effects	34% [21%; 47%]	Tau^2 = 0.0523; Chi^2 = 306.71, df = 11 (*P* < 0.01); I^2 = 96%
	3–6 months (Fig. 6B)	4	4	2297	Random Effects	15% [5%; 26%]	Tau^2 = 0.0109; Chi^2 = 22.48, df = 3 (*P* < 0.01); I^2 = 87%
	6–12 months (Fig. 6C)	11	10	1343	Random Effects	13% [8%; 17%]	Tau^2 = 0.0038; Chi^2 = 30.08, df = 10 (*P* < 0.01); I^2 = 67%
	> 12 months (Fig. 6D)	36	34	3453	Random Effects	10% [8%; 13%]	Tau^2 = 0.0041; Chi^2 = 311.79, df = 35 (*P* < 0.01); I^2 = 89%
	Overall	63	45	5093	Random Effects	16% [12%; 19%]	Tau^2 = 0.0182; Chi^2 = 991.31, df = 62 (*P* < 0.01); I^2 = 94%
Prevalence of TSH deficiency (Fig. 7)	< 3 months (Fig. 7A)	14	11	1184	Random Effects	11% [7%; 15%]	Tau^2 = 0.0051; Chi^2 = 66.35, df = 13 (*P*< 0.01); I^2 = 80%
	3–6 months (Fig. 7B)	5	5	457	RE	3% [1%; 6%]	Tau^2 = 0.0004; Chi^2 = 8.51, df = 4 (*P* = 0.07); I^2 = 53%
	6–12 months (Fig. 7C)	11	10	658	RE	4% [1%; 6%]	Tau^2 = 0.0010; Chi^2 = 28.4, df = 10 (*P* < 0.01); I^2 = 65%
	> 12 months (Fig. 7D)	31	31	2763	RE	5% [3%; 7%]	Tau^2 = 0.0022; Chi^2 = 160.54, df = 30 (*P* < 0.01); I^2 = 81%
	Overall	61	45	5062	RE	6% [5%; 7%]	Tau^2 = 0.0024; Chi^2 = 285.98, df = 60 (*P* < 0.01); I^2 = 79%

#### Growth hormone (GH) deficiency

The analysis encompassed 64 datasets and 46 studies, totaling 5292 participants. The pooled estimate for GH deficiency prevalence was 18% (95% CI [14%; 21%]). Subgroup analysis based on duration revealed varying prevalence rates: 18% (95% CI [11%; 24%]) for < 3 months, 13% (95% CI [5%; 22%]) for 3–6 months, 11% (95% CI [7%; 16%]) for 6–12 months, and 19% (95% CI [14%; 24%]) for > 12 months. Heterogeneity levels were substantial, ranging from 68 to 94%, as shown in Fig. 4.

#### Adrenocorticotropic hormone (ACTH) deficiency

For ACTH deficiency, the analysis included 66 datasets and 47 studies with a total of 5376 participants. The overall prevalence was 10% (95% CI [8%; 13%]). Subgroup analysis by duration showed prevalence rates of 7% (95% CI [3%; 11%]) for < 3 months, 12% (95% CI [2%; 22%]) for 3–6 months, 5% (95% CI [2%; 7%]) for 6–12 months, and 12% (95% CI [8%; 16%]) for > 12 months. Heterogeneity varied substantially across durations, ranging from 75 to 94%, as depicted in Fig. 5.

#### Pituitary–Gonadal axis hormones deficiency

The analysis for pituitary–gonadal axis hormones deficiency involved 63 datasets and 45 studies, with 5093 participants. The pooled prevalence was 16% (95% CI [12%; 19%]). Prevalence rates differed across durations: 34% (95% CI [21%; 47%]) for < 3 months, 15% (95% CI [5%; 26%]) for 3–6 months, 13% (95% CI [8%; 17%]) for 6–12 months, and 10% (95% CI [8%; 13%]) for > 12 months. Notably, heterogeneity was high, ranging from 67 to 96%, as illustrated in Fig. 6.

#### Thyroid-Stimulating hormone (TSH) deficiency

The analysis of TSH deficiency included 61 datasets and 45 studies, totalling 5062 participants. The overall prevalence was 6% (95% CI [5%; 7%]). Prevalence rates by duration were 11% (95% CI [7%; 15%]) for < 3 months, 3% (95% CI [1%; 6%]) for 3–6 months, 4% (95% CI [1%; 6%]) for 6–12 months, and 5% (95% CI [3%; 7%]) for > 12 months. Heterogeneity ranged from 53 to 81%, as shown in Fig. 7.

These findings highlight the variability in prevalence rates of specific axes affection post-TBI across different durations, emphasising the need for tailored clinical management strategies based on the duration and type of pituitary axis dysfunction observed.

## Discussion

TBI represents a significant public health concern globally, with long-term consequences extending beyond the initial injury [1, 2]. One such consequence that has garnered increasing attention is hypopituitarism, characterised by pituitary axis dysfunction. The pituitary gland plays a crucial role in regulating hormone production, and disruption post-TBI can lead to a range of endocrine abnormalities [6, 7]. However, the prevalence and temporal patterns of pituitary axis dysfunction following TBI have not been comprehensively elucidated, prompting this systematic review and meta-analysis to provide a more nuanced understanding of this complex relationship.

Our meta-analysis synthesised data from 52 studies comprising 7367 participants, shedding light on the prevalence and temporal changes in pituitary axis dysfunction post-TBI. The pooled prevalence of any axis affection was found to be 33% (95% CI [28%; 37%]), indicating a substantial burden of pituitary dysfunction in this population. Subgroup analysis based on duration post-TBI revealed intriguing temporal patterns. Within the first 3 months, the prevalence of any axis affection peaked at 40% (95% CI [27%; 53%]), gradually decreasing over time to 31% (95% CI [15%; 47%]) at 3–6 months, 26% (95% CI [19%; 33%]) at 6–12 months, and 32% (95% CI [26%; 38%]) beyond 12 months. These findings suggest an initial surge in pituitary dysfunction post-TBI, followed by a gradual decline, although prevalence remains elevated even in the chronic phase.

In terms of multiple axes affection, our analysis revealed an overall prevalence of 7% (95% CI [6%; 9%]), indicating that while simultaneous dysfunction across multiple axes is less common, it is still clinically significant. Subgroup analysis by duration showed relatively stable prevalence rates across different timeframes, ranging from 13% (95% CI [6%; 20%]) for < 3 months to 7% (95% CI [5%; 9%]) for > 12 months.

The observed prevalence rates of any axis affection post-TBI align with previous literature documenting a high prevalence of pituitary axis dysfunction in this population [7, 9]. The initial surge in dysfunction within the first 3 months could be attributed to acute TBI-related pathophysiological processes, such as neuroinflammation post-TBI trigering an immune response that aims to clear damaged neuronal cells which can become prolonged or excessive, leading to secondary damage, as well as neuroendocrine disruption due to direct damage to the hypophysis axes. As the post-TBI period progresses, a combination of adaptive mechanisms and therapeutic interventions may contribute to the gradual decline in prevalence, although persistent dysfunction underscores the chronic nature of this complication [70, 71].

Comparing our findings with existing literature, several studies have reported comparable prevalence rates of pituitary dysfunction following TBI. For example, Agha et al. (2005) found a prevalence of 35% for any axis affection, corroborating our overall estimate. Similarly, Aimaretti et al. (2004) [18] and Bondanelli et al. (2004) [26] highlighted the dynamic nature of pituitary dysfunction post-TBI, with prevalence rates mirroring our subgroup analyses based on duration [72, 73].

The stability of multiple axes affection prevalence across different timeframes suggests that while initial dysfunction may involve multiple axes, the chronic phase often manifests as isolated or fewer axis dysfunctions. This observation is supported by studies such as Berg et al. (2010), which demonstrated a shift in pituitary axis involvement over time post-TBI [8, 9, 25].

Notably, specific axes affection showed distinct prevalence rates, with GH deficiency being the most prevalent at 18% (95% CI [14%; 21%]). This finding is consistent with prior research highlighting GH deficiency as a common consequence of TBI-induced hypopituitarism. Studies emphasised the clinical significance of GH deficiency in TBI patients, underscoring the importance of targeted screening and management strategies [10].

Similarly, the prevalence of ACTH deficiency (10%; 95% CI [8%; 13%]), pituitary–gonadal axis hormones deficiency (16%; 95% CI [12%; 19%]), and TSH deficiency (6%; 95% CI [5%; 7%]) provides valuable insights into the spectrum of pituitary axis involvement post-TBI. These findings resonate with previous literature elucidating the multifaceted endocrine disturbances following TBI, as highlighted by studies. Clinical manifestation of the deficiencies is presented in (Table 4) [6, 10].

**Table 4:** Clinical Implications of Pituitary Hormone Deficiencies

Hormonal Deficiency	Symptoms	Clinical Findings
*GH*	- Fatigue - Decreased muscle mass - Increased body fat - Depression or anxiety - Poor quality of life - Reduced exercise capacity	- Low GH levels on stimulation test - Reduced IGF-1 levels - Decreased lean body mass - Increased fat mass
*LH/FSH*	- Reduced libido - Infertility - Irregular or absent menstruation (in women) - Erectile dysfunction (in men) - Hot flashes	- Low testosterone levels (men) - Low estrogen levels (women) - Low or inappropriately normal LH/FSH levels
*ACTH*	- Fatigue - Weight loss - Nausea, vomiting - Dizziness, especially upon standing - Low blood pressure	- Low cortisol levels - Low ACTH levels - Hyponatremia (low sodium) - Hypoglycemia (low blood sugar)
*TSH*	- Fatigue - Weight gain - Cold intolerance - Constipation - Dry skin, hair loss - Depressed mood	- Low free T4 and T3 levels - Low or inappropriately normal TSH levels - Bradycardia (slow heart rate) - Elevated cholesterol levels

### Gaps and limitations

The study has several limitations. First, potential risk factors related to TBI, particularly those involving clinical manifestations in the acute phase and conditions that mimic hypopituitarism, remain unclear and require further investigation. Second, there is a possibility of publication bias, as studies with positive findings are more likely to be published, which may have distorted the results. Third, by restricting the search to English-language studies, significant research published in other languages may have been excluded, which could impact the generalizability of the findings. Additionally, incomplete reporting in some studies posed challenges for data extraction and quality assessment. Lastly, despite employing a comprehensive search strategy, some relevant recent studies may have been overlooked.

## Conclusion

In conclusion, our meta-analysis underscores the high prevalence and dynamic nature of pituitary axis dysfunction following TBI, with distinct temporal patterns and axis-specific variations. Key findings include a pooled prevalence of any pituitary axis affection at 33% (95% CI [28%; 37%]), underscoring the substantial burden of pituitary dysfunction in this population. Growth hormone (GH) deficiency was found to have the highest pooled prevalence among all axes (18% (95% CI [14%; 21%])), with the highest rates observed beyond 12 months post-injury at 19% (95% CI [14%; 24%]). Adrenocorticotropic hormone (ACTH) deficiency showed an overall prevalence of 10% (95% CI [8%; 13%]), with rates peaking at 12% (95% CI [2%; 22%]) during the 3–6 months post-injury. Pituitary–gonadal axis hormone deficiency exhibited a pooled prevalence of 16% (95% CI [12%; 19%]), with a particularly high prevalence within the first three months at 34% (95% CI [21%; 47%]). Thyroid-stimulating hormone (TSH) deficiency had the lowest overall prevalence at 6% (95% CI [5%; 7%]), with a peak within the first three months at 11% (95% CI [7%; 15%]). These findings highlight the persistent and varying nature of pituitary dysfunction following TBI, emphasizing the need for ongoing monitoring and tailored clinical management of affected patients. The variability in prevalence rates raises the question of how extensive is the role played by factors such as TBI severity, patient demographics, and both clinical and paraclinical variables in this inconsistency, highlighting the need for further research to clarify these relationships.

## Data Availability

No datasets were generated or analysed during the current study.
